# Formulation of Poloxamers for Drug Delivery

**DOI:** 10.3390/jfb9010011

**Published:** 2018-01-18

**Authors:** Andrew M. Bodratti, Paschalis Alexandridis

**Affiliations:** Department of Chemical and Biological Engineering, University at Buffalo, The State University of New York (SUNY), Buffalo, NY 14260, USA; bodratti@buffalo.edu

**Keywords:** Pluronic, poly(ethylene oxide), poly(ethylene glycol), nanomedicine, excipient, formulation, solubilization, anticancer, micelle, nanoparticle, hydrogel

## Abstract

Poloxamers, also known as Pluronics^®^, are block copolymers of poly(ethylene oxide) (PEO) and poly(propylene oxide) (PPO), which have an amphiphilic character and useful association and adsorption properties emanating from this. Poloxamers find use in many applications that require solubilization or stabilization of compounds and also have notable physiological properties, including low toxicity. Accordingly, poloxamers serve well as excipients for pharmaceuticals. Current challenges facing nanomedicine revolve around the transport of typically water-insoluble drugs throughout the body, followed by targeted delivery. Judicious design of drug delivery systems leads to improved bioavailability, patient compliance and therapeutic outcomes. The rich phase behavior (micelles, hydrogels, lyotropic liquid crystals, etc.) of poloxamers makes them amenable to multiple types of processing and various product forms. In this review, we first present the general solution behavior of poloxamers, focusing on their self-assembly properties. This is followed by a discussion of how the self-assembly properties of poloxamers can be leveraged to encapsulate drugs using an array of processing techniques including direct solubilization, solvent displacement methods, emulsification and preparation of kinetically-frozen nanoparticles. Finally, we conclude with a summary and perspective.

## 1. Introduction

Poloxamers, available also under the trademark Pluronics^®^ (BASF), are a class of water-soluble nonionic A-B-A and B-A-B triblock copolymers, where A is poly(ethylene oxide) (PEO) and B is poly(propylene oxide) (PPO). Tetronic^®^ block copolymers, also offered by BASF, comprise four PPO-PEO chains, which extend outward from an amine-terminated central chain [1,2,3]. While traditional surfactants are low molecular weight, block copolymers are long chains (several thousand Da). The monomers comprising the copolymer blocks are chemically dissimilar (e.g., polar and non-polar), rendering the block copolymers amphiphilic and leading to surface active properties. The block segregation gives rise to interesting and useful nanostructures, which are spontaneously formed in solution (self-assembly). Poloxamers exhibit an amphiphilic character in aqueous solution on the basis of the PEO solubility in water and the PPO insolubility. The PEO blocks are thus hydrophilic, while the PPO block is hydrophobic.

The size and structure of poloxamer assemblies, and their adsorption properties [4,5], have made them useful in many applications, including: Drug delivery [6], nanoparticle synthesis [7], cosmetics [8] and emulsion [9] formulation, effective dispersants for inks/pigments [10] and as versatile anti-biofouling coatings [11], to name a few. The use of poloxamers in pharmaceutical research is widely researched. Recent reviews have covered various drug delivery forms (thermosensitive gels [12,13,14,15,16], cubosomes [17], micelles [18,19,20]), specific applications (ophthalmic [21], oral chemotherapy [22], lung cancer treatment [23], gene delivery [24,25]) and multi-drug resistance (MDR) reversal [26,27,28]. Questions surrounding the effective delivery of nanomedicines, especially anticancer drug delivery to tumors, have come to light recently [29]. While nano-sized drug vehicles may benefit from the enhanced permeability and retention (EPR) effect, where therapeutics can enter tumors via leaky blood vessels, median delivery efficiencies remain less than 1% [29]. This motivates further work in the development of drug carriers with better targeting.

The aim of this review is to connect the self-assembly properties of poloxamers to their utility in formulating therapeutic delivery systems and in particular how they make poloxamers amenable to multiple processing routes that serve specific needs. The mechanisms that enable poloxamer drug delivery are responsible for advances in a continually expanding number of treatments, each with their own unique challenges. Recent reviews that address poloxamers have delved into specific structures (e.g., hydrogels [30], core-shell vehicles [31], poloxamer-modified hydrophobic particles [32]) or disease/administration routes (e.g., anticancer [33], ophthalmic [21]). Analysis of recent literature (published over the past five years) covering a range of treatment pathways and diseases reveals a major emphasis on “smart” drug carriers developed with poloxamers. The diverse range of potential delivery methods is highlighted in this review by discussing how the poloxamer solution behavior enables multiple formulation processing routes, drug-encapsulating structures, and engagement with physiological barriers to drug passage. Processing-structure-property relationships for poloxamer formulations are emphasized due to their important contribution to drug delivery mechanisms. Insights into the underlying mechanisms described may be useful to formulators. Important parameters such as drug release rate and formulation stability are directly linked to poloxamer associative and adsorption properties. There is a large number of functional block copolymers being evaluated for drug carriers [34,35,36]. Some of these are responsive to stimuli including ultrasound and pH, depending on the target. Many of these include PEO (frequently denoted as PEG) due to its usefulness as a stabilizer. Poloxamers are a convenient choice for formulators due to their commercial availability, wide range of molecular weights and compositions and, for some poloxamers, approval for use in pharmaceuticals, enabling direct translation from fundamental research to clinical applications. Chemically-modified poloxamers, e.g., with polyacrylic acid, which confers pH sensitivity, have also been investigated [37,38,39].

What follows is a discussion of how poloxamers can be processed to create tailored delivery systems, including an overview of common processing techniques. Before this, we begin with a general description of the self-assembly properties of poloxamers in aqueous solutions, which are central to the formulation methods. The first processing method discussed is direct solubilization, one of the simplest ways to encapsulate drug in thermodynamically-stable micelles. Next, methods that rely on organic solvents to modulate the poloxamer structure and drive encapsulation are presented. Two of these, thin film hydration and temperature-induced emulsification, rely on temperature changes to create drug-loaded phases. Finally, a generalized method is reviewed to prepare poloxamer-stabilized drug-loaded nanoparticles with high drug-to-excipient ratios.

## 2. Poloxamer Self-Assembly in Aqueous Solutions

Poloxamers are available in a broad range of molecular weights and PPO/PEO ratios. Table 1 shows examples of commercially available Pluronics^®^. BASF utilizes a specific notation for Pluronics^®^, where the first letter indicates the physical state (P: Paste, F: Flake, L: Liquid), the first one or two numbers relate to the molecular weight and the last number indicates the weight percent of the PEO block [40]. The aqueous solution properties of poloxamers have been intensely studied and thoroughly reviewed [1,2,3,40,41,42] owing to their unique behavior and benefit to myriad applications. The associative properties of poloxamers in the most common solvent, water, are discussed here.

Poloxamer molecules form an array of thermodynamically-stable self-assembled structures in solution, driven by differences in the solubility of their constituent PEO and PPO blocks. Individual non-associated block copolymer chains are often termed unimers, to distinguish them from chains, which are organized into supramolecular structures. Since solubility drives self-assembly, the solvent type and temperature are important in determining the system properties. The impacts on self-assembly of block copolymer concentration, molecular weight and PPO/PEO ratio, along with solvent quality, have been explored in many binary [43,44] (water + poloxamer) and ternary (water + “oil” + poloxamer) systems [45,46,47]. Observed self-assembled structures include micelles, reverse (water-in-oil) micelles and lyotropic liquid crystals (LLC), including lamellar (stacked layer-like assemblies), hexagonal (cylinder-like assemblies) and bicontinuous or micellar cubic [47].

The association in aqueous solution of poloxamer unimers into micelles is well understood. The micellization process commences when the PEO-PPO block copolymer concentration in solution reaches a certain critical micellization concentration (cmc) at a fixed temperature. Conversely, increasing the temperature to the critical micellization temperature (cmt) at a fixed block copolymer concentration also induces micellization. The temperature effect arises from the reduced solubility of the PEO, and especially the PPO, blocks in water upon heating [48]. This thermo-responsive feature of poloxamer self-assembly has been used to significant effect in drug delivery. The temperature dependence of micellization can be interpreted and predicted by the use of a two-state model, which considers both polar and apolar interactions in the PEO and PPO segments [49]. The result of the reduced solubility is a microphase separation of the PPO blocks out of the aqueous environment and into the micelle core. Micellization of poloxamers, and amphiphiles in general, is an equilibrium process. This being the case, the temperature effect on poloxamer micellization is fully reversible. While the PEO and PPO blocks covalently joined to a poloxamer polymer chain (block copolymer) enable self-assembly behavior important to the solubilization of drugs, PEO and PPO chains individually (homopolymers) are not capable of such organization. However, homopolymer PEO is useful in drug delivery for surface modification, the stabilization of colloids and as an excipient to form solid particles [50].

The microphase separation that results in thermodynamically-stable micelles involves two processes [51]. The first process is fast (hundredths-to-tenths of milliseconds) and represents unimers assembling into metastable aggregates. The second process is a relaxation of the aggregates to thermodynamically-stable micelles and is slower, on the order of 1–100 ms. Comprehensive studies of cmc and cmt data for poloxamers in aqueous solution highlight the relationship between block copolymer molecular characteristics and their solution association and surface adsorption properties [52,53]. Poloxamers with long PPO blocks require lower block copolymer concentrations or temperatures for micellization to occur. For poloxamers with the same PPO/PEO ratio, both the cmc and cmt decrease as the overall molecular weight increases. This effect is stronger for poloxamers with higher relative PPO content [53].

Micellization is a spontaneous process, which is attractive in a practical sense, as it simplifies processing in formulated products. In the case of poloxamers, micellization is highly endothermic (several hundreds of kJ/mol), owing to the bending and breaking of hydrogen bonds between water molecules in order to accommodate the PEO-PPO-PEO unimers in solution. When unimers associate into micelles, water molecules become less constrained, and the entropy of the system is increased. The positive entropy term more than compensates for the high enthalpy, leading to an overall negative Gibbs free energy of micellization. Poloxamer micellization is thus said to be entropically driven [53].

**Figure 1:**
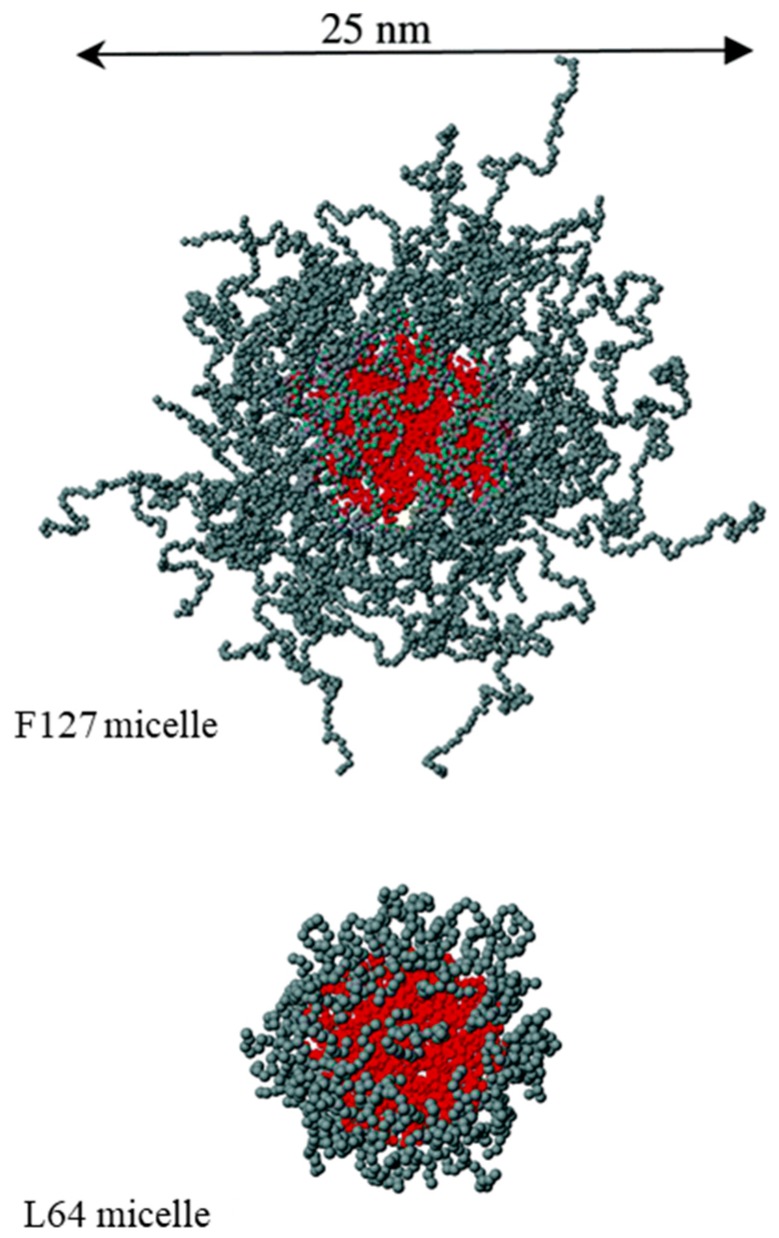
Configurations of Pluronic^®^ F127 (EO_100_PO_65_EO_100_) and L64 (EO_13_PO_30_EO_13_) micelles determined from coarse-grained implicit solvent simulations. The micelle core is made up of the PPO blocks, while the corona is formed from the PEO blocks. Reprinted with permission from [55]. Copyright 2006 American Chemical Society.

Generally, poloxamer micelles in aqueous solution are spherical with a core-shell structure (Figure 1) [54,55]. Fluorescence probe studies using pyrene show that the micelle core is hydrophobic and dominated by PPO blocks, while the corona, in contact with the bulk aqueous environment, is comprised of hydrated PEO blocks [56]. The heat-induced micellization process has been observed to take place over a range of about 5 °C, attributable to polydispersity in poloxamer systems. As the temperature is increased beyond the cmt, the aggregation number (i.e., average number of block copolymer chains comprising one micelle, N_agg_) increases, while the overall micelle hydrodynamic radius remains approximately constant [56,57]. The hydrodynamic radius corresponds to the equivalent hard sphere model radius. Small-angle neutron scattering (SANS) studies of micellar Pluronic^®^ L64 (EO_13_PO_30_EO_13_) showed that the N_agg_ increased from 37–54 when the temperature was increased from 37.5–55 °C [58]. At the same time, the (PPO-rich) core radius did not change in size and in fact became further dehydrated and dense, while the overall micelle size grew slightly (from 50–56 nm). The reason for the increase in N_agg_ is dehydration of the PEO blocks, which allows for packing of more polymer chains into the micelle.

**Figure 2:**
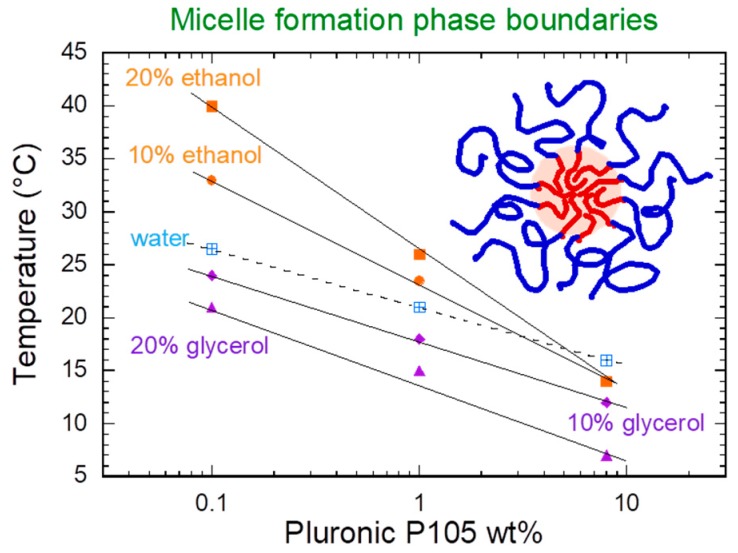
Micellization boundaries for aqueous Pluronic^®^ P105 (EO_27_PO_56_EO_27_) solutions with added polar organic solvents. Micelles form at poloxamer concentrations and temperatures above the lines. Note that the micellization boundaries shift depending on the quality of the solvent. Data obtained from [69].

Poloxamer PEO-PPO-PEO block copolymers in aqueous solution exhibit a rich polymorphism with changes in temperature and concentration [43,44,54]. Micelles of Pluronic^®^ P85 (EO_27_PO_39_EO_27_) have been observed to transition from spheres to rods [59,60] and prolate ellipsoids [61] at high temperature (around 70 °C). Additives such as polar organic solvents [62,63,64,65,66], urea [67] and salts [68] are known to influence micellization properties and micelle structure by changing the solvent quality. As shown in Figure 2, the micellization boundary for Pluronic^®^ P105 (EO_27_PO_56_EO_27_) can be drastically influenced by the addition to water of cosolvents, which alter the amount of hydration available for the PEO and PPO blocks [69]. Properties such as the diffusion coefficient and solution viscosity change markedly upon micelle shape transitions [59].

**Figure 3:**
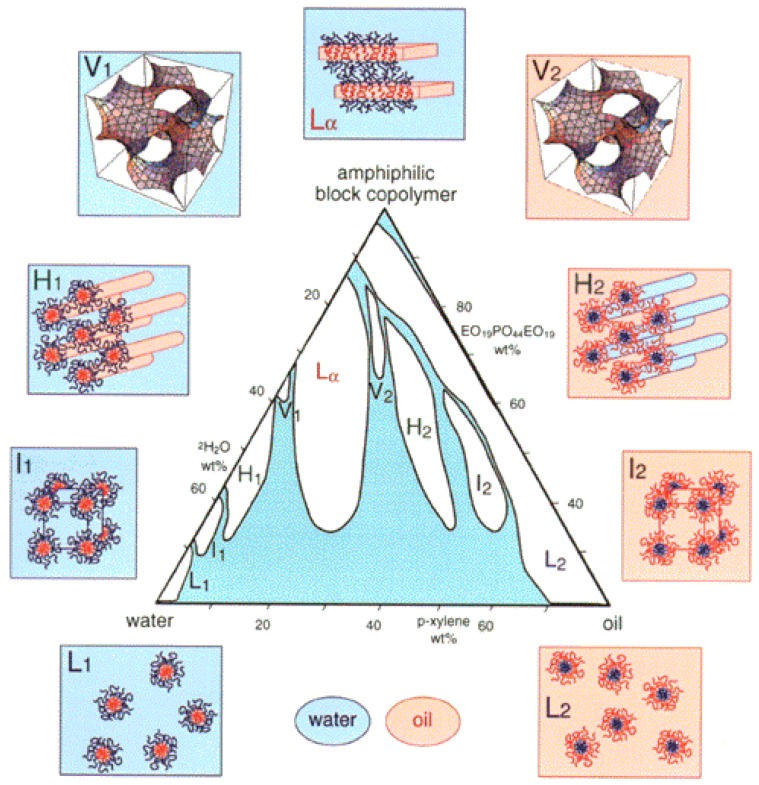
Ternary isothermal phase diagram of Pluronic^®^ P84 (EO_19_PO_43_EO_19_) in water and *p*-xylene at 25 °C. l_1_, H_1_, V_1_, Lα, V_2_, H_2_ and l_2_ indicate normal (“oil-in-water”) micellar cubic, normal hexagonal, normal bicontinuous cubic, lamellar, reverse (“water-in-oil”) bicontinuous cubic, reverse hexagonal and reverse micellar lyotropic liquid crystalline phases, respectively. L_1_ and L_2_ indicate normal micellar and reverse micellar solutions. Reprinted with permission from [47]. Copyright 1998 American Chemical Society.

The formation of thermo-reversible “gels” at higher block copolymer concentrations and temperatures has also been reported for several poloxamers [43,54,70,71]. The “gels,” which are clear and stiff, result from a phase transition from the micellar solution state to a solid micellar cubic (typically bcc structure) state. The phase transition takes place as the volume fraction occupied by hydrated micelles (φ) increases to that required for hard-sphere crystallization (φ_c_ = 0.53), resulting in micelle-micelle interactions and entanglements [54,71]. Several poloxamers have been observed to form micellar cubic LLC structures, also known as hydrogels, including Pluronic P84 (EO_19_PO_43_EO_19_) and Poloxamer 407 (EO_100_PO_65_EO_100_). Poloxamer 407 is widely used because it can form hydrogels at lower block copolymer concentrations in water, around 20% *w*/*w*. Micelles of Poloxamer 407 will have extended, well-hydrated outer shells of PEO, which rapidly leads to a closely-packed organization of the micelles above the cmt. The elastic and viscous moduli of aqueous Poloxamer 407 increase several orders of magnitude as the solution transitions from a micellar to a micellar cubic LLC structure (hydrogel), indicating a sturdy gel [72]. Several other lyotropic liquid crystalline phases have been discovered in ternary poloxamer-water-oil isothermal systems, including normal and reverse cubic and hexagonal LLC phases (Figure 3) [46,47,73,74,75,76,77,78]. Poloxamers can also form mixed micelles in the presence of added surfactants, resulting in different cmc values [79,80].

The ability of a given poloxamer block copolymer to form one of a number of different thermodynamically-stable (reversible) structures depending on concentration, temperature and cosolvents, is one of their most unique and powerful properties. In the context of drug delivery, this lends a significant number of design options to formulators working with a specific drug, disease and delivery route. Micelles with various structures (i.e., ellipsoids, rods) are thermodynamically stable. As micelles of different shapes become closely packed, care must be taken to monitor drug release rates. Pluronic P123, for example, can form cylindrical micelles, which stack together, leading to decreased release rates [81]. LLC phases are equilibrium structures, which, due to their close packing of assemblies, are often kinetically stable and mechanically robust. The structures can be tuned by modulating the temperature or adding cosolvents [43,46,76,78]. The influence of a cosolvent is derived from its selective solubility for one block or the other and its impact on the bulk solvent structure. Polar organic solvents, salts, urea and glucose, for example, lead to changes in micelle shapes for poloxamers dissolved in water by altering the hydration of the PEO and PPO blocks. Less polar solvents (“oil”), as in ternary block copolymer-oil-water isothermal systems, work by swelling the constituent blocks to different extents, depending on the relative amounts of the two solvents, thereby altering the interfacial curvature to produce different structures [82].

## 3. Preparation of Poloxamer-Based Drug Delivery Formulations

The low aqueous solubility of hydrophobic drugs is a limiting factor in pharmaceutical design (Table 2). Several methodologies that exploit the self-assembly and solubilization properties of poloxamers have been investigated to circumvent this problem. Poloxamers are amenable into several types of processing because of their high sensitivity to temperature and other self-assembly properties. For a given final composition, different processing routes may result in different physicochemical properties. This is true of poloxamer emulsions, which display changes in solubilization capacity, viscosity and long-term stability, depending on the emulsification pathway [83].

The simplest method for preparing drug-loaded poloxamer-based formulations, direct solubilization, is described first. Methods requiring heating to achieve the final product structure are presented next. Following this, so-called solvent displacement methods, which take advantage of poloxamer behavior in mixed solvent systems, are discussed. Finally, a methodology that utilizes the cmc-switching behavior of poloxamers to produce kinetically-frozen micelles is described. While the following sections emphasize the mechanisms by which poloxamers facilitate drug solubilization, poloxamers are also useful for colloidal stabilization because they can adsorb on both hydrophobic and hydrophilic surfaces, producing a brush in doing so [4]. We do not discuss this generally here, but there are several examples in the following sections that illustrate how poloxamers stabilize the formulated structure. This is especially the case in emulsions, cubosomes and frozen micelles, where steric stabilization is required.

### 3.1. Direct Solubilization of Actives

As discussed, poloxamers will form spherical micelles in aqueous solution spontaneously at the cmc (or cmt). The PPO-rich hydrophobic domains thus formed are capable of accommodating drugs via solubilization. The phenomenon of solubilization can be described by thermodynamic concepts similar to those applied to explain dilute solution micellization. Specifically, a favorable change in free energy occurs when a hydrophobic solute is localized within the PPO blocks of the micelle core [86,87,88]. Drug-block copolymer interactions thus represent a degree of freedom to consider in formulation, since they influence the cmc or cmt. Organic solvents [63] and salts [68], often included as excipients and to aid drug solubility, also influence the cmc and micelle shape.

The poloxamer PPO/PEO ratio, number of EO (N_EO_) and PO (N_PO_) units and overall molecular weight control physicochemical properties including the cmc, cmt and micelle aggregation number (N_agg_). These in turn relate directly to the solubilized amount of drug and the release profile. Generally, poloxamers with a larger number of PO units result in higher partition coefficients [89], defined as the amount of hydrophobic solute in the micellar phase compared to that in the aqueous phase. Recent investigations of how poloxamer molecular composition relates to drug loading have found that both the PPO/PEO ratio and the polymer molecular weight are crucial [90,91,92]. Pluronics^®^ P103 (EO_17_PO_60_EO_17_) and P123 (EO_19_PO_69_EO_19_) showed a higher capacity to solubilize three moderately hydrophobic drugs (genistein (GEN), paclitaxel (PCL) and quercetin (QCT)) than Pluronic^®^ F127, despite being roughly half the molecular weight [90]. Both P103 and P123 have higher PPO/PEO ratios (1.82 and 1.80 for P103 and P123, respectively) compared with F127 (0.34). In another example, the solubilization of fenofibrate, which is sparingly soluble in water, is enhanced by poloxamers with more PO units. Poloxamers P9200, P10300, P10400 and P10500 (having 50, 50, 61 and 56 PO units, respectively) showed an over 100-fold increase in the solubilization of fenofibrate at 5 wt % poloxamer and 25 °C [92].

Solubility enhancement is suppressed in poloxamer block copolymers with longer PEO blocks due to the weak interaction between the polar PEO and the hydrophobic drug. However, the thick PEO brush, which forms the micelle corona, also impedes the transport of drug into the micelle core [93,94]. Systematic solubilization studies using Pluronic^®^ P84 (EO_19_PO_43_EO_19_), F127 (EO_100_PO_65_EO_100_) and F108 (EO_132_PO_50_EO_132_) showed that block copolymers with longer PEO blocks incorporated less drug at poloxamer concentrations between 0.1 and 5 *w*/*v* %, with the disparity growing with increasing poloxamer concentration [93]. In some cases, such as Pluronic^®^ L43 (EO_13_PO_22_EO_13_), the block copolymer may simply possess too small a molecular weight to store the drug or to form micelles at a convenient processing temperature [91].

**Figure 4:**
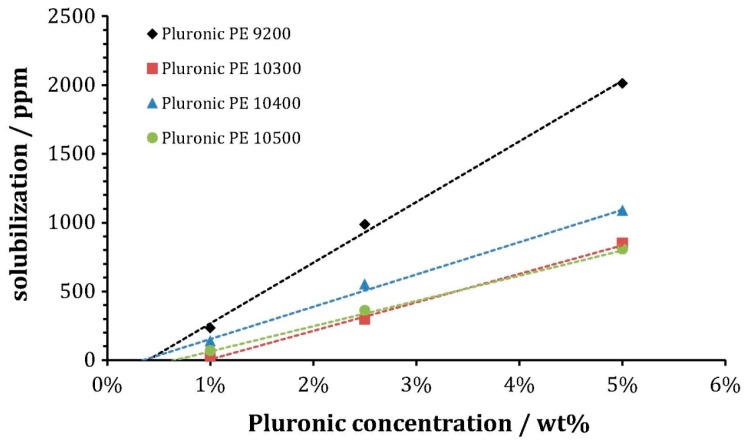
The amount of hydrophobic fenofibrate, a cholesterol reducer, solubilized in aqueous poloxamer solution increases linearly with block copolymer concentration. The fenofibrate was added to micellar aqueous poloxamer solutions and allowed to equilibrate for 24 h at 25 °C. Pluronic^®^ PE 9200 (EO_8_PO_47_EO_8_), 10300 (EO_16_PO_56_EO_16_), 10400 (EO_25_PO_56_EO_25_) and 10500 (EO_37_PO_56_EO_37_). Reprinted from [92], Copyright 2016, with permission from Elsevier.

The amount of drug solubilized in poloxamer micelles increases linearly with the block copolymer concentration [92], as shown in Figure 4, which depicts the solubilized amount of hydrophobic fenofibrate with poloxamer concentration as detected by UV-Vis spectroscopy. The encapsulation efficiency, defined as the ratio of the weight of drug in micelles to the total weight of the drug in the formulation, increases sharply and non-linearly with concentration. For example, the encapsulation efficiency of oxcarbazepine into aqueous Pluronic^®^ P84 changed from 19.16–24.3% as the P84 concentration increased from 1–2% *w*/*v*, but changed from 52.36–98.33% as it increased from 4–5% *w*/*v* [93]. The total solubilization capacity of a thermodynamically-stable micelle is defined by free energy contributions [95,96,97,98]. The finite capacity is driven strongly by the block copolymer structure: a high number of unfavorable EO-(hydrophobic) solute interactions occur in micelles with a small PPO core, resulting in the solubilization of fewer molecules.

The drug polarity plays an important role in determining the solubilized amount (and steepness of the solubilization curve), as it dictates the strength of interactions with the PEO and PPO blocks. In the case of carbamazepine, nuclear Overhauser effect spectroscopy (NOESY)-NMR experiments indicated that the drug protons interacted with both the PEO and PPO protons, suggesting a non-specific association with the block copolymer [92]. On the other hand, protons from the more hydrophobic drug fenofibrate interacted exclusively with PPO. As a result, a stronger enhancement in solubility was observed, especially under conditions where micellization occurred [92].

**Figure 5:**
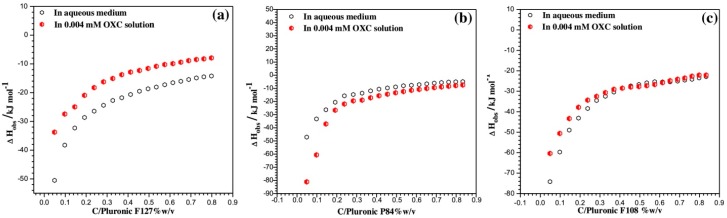
ITC curves generated from ITC experiments for the titration of different poloxamers ((**a**) Pluronic F127; (**b**) Pluronic P84; (**c**) Pluronic F108) into water (empty symbols) and aqueous 0.004 mM oxcarbazepine (OXC) solutions (filled symbols) at 37 °C. OXC is used to reduce the frequency of epileptic occurrences. The micellization process was endothermic for all poloxamers in both solutions. The presence of OXC made the micellization of Pluronic F127 (EO_100_PO_65_EO_100_) and F108 (EO_132_PO_50_EO_132_) less endothermic due to its interaction with the long PEO blocks. The micellization process was more endothermic in Pluronic^®^ P84 (EO_19_PO_43_EO_19_) solutions, owing to strong hydrophobic interactions with the PPO block. Reprinted from [93], Copyright 2016, with permission from Elsevier.

**Figure 6:**
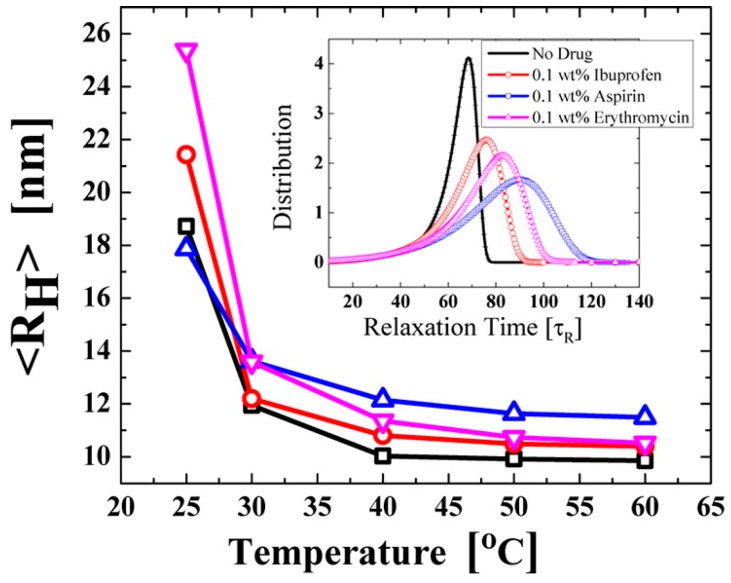
Dynamic light scattering measurement of aqueous 5.26 wt % F127-drug systems: no drug (squares), 0.1 wt % ibuprofen (circles), 0.1 wt % aspirin (up-triangles) and 0.1 wt % erythromycin (down-triangles). The drug was added to micellar F127 solutions by vigorous mixing at temperatures between 40 and 60 °C. The average hydrodynamic radius (R_H_) of the micelles decreased sharply with temperature at first. Aspirin, the least hydrophobic drug, led to the formation of the largest and most polydisperse micelles. The distribution of relaxation times, which were calculated from the autocorrelation scattering functions from the DLS measurement, is shown in the inset: no drugs (solid line), 0.1 wt % ibuprofen (circle-line), 0.1 wt % aspirin (square-line) and 0.1 wt % erythromycin (triangle-line). Wide distributions indicate high polydispersity. Ibuprofen, the most hydrophobic of the evaluated drugs, produced the most compact structures, while it resulted in the least polydisperse micelles. Reprinted with permission from [99]. Copyright 2013 American Chemical Society.

While the hydrophobic effect is responsible for micellization in aqueous PEO-PPO-PEO systems [53], interactions between the block copolymer and added drug can alter the cmc, cmt and other physicochemical properties. The change in solution properties caused by addition of drug is a useful indicator for the strength of such interactions. For example, the addition of 0.6 wt % flurbiprofen to solutions of Pluronic^®^ P123 in 10% ethanol-water solutions decreased the cmc from 0.029 to 0.007 *w*/*v* % at 25 °C [91], indicating strong cooperativity between P123 and the drug. In an analogous manner, the cmt of Pluronic^®^ F127 (5.26 wt %) decreased from 23 °C to less than 12 °C in the presence of just 0.1 wt % ibuprofen [99]. A cmt reduction has also been observed in systems such as P104-ibuprofen [100] and mixed F127-F68 with lidocaine [101], among others. Isothermal titration calorimetry (ITC) allows for direct measurement of the enthalpy of interactions between molecules, e.g., poloxamer with a drug. This allows for accurate calculation of the thermodynamic properties of interest for formulation, such as cmc and enthalpy of micellization [102,103]. Enthalpograms for the titration of Pluronics^®^ F127, F108 and P84 (all 5% *w*/*v*) into 0.004 mM oxcarbazepine solution gave insight to the nature of the drug-block copolymer interaction (Figure 5). In the case of hydrophilic block copolymers (F127 and F108), the mixing of micelles with drug is less endothermic than mixing in plain water, indicating stronger interaction of oxcarbazepine with the PEO corona. Pluronic^®^ P84 showed the opposite case (more endothermic in the presence of drug), owing to the solubilization of the drug into the core, resulting in PPO dehydration from hydrophobic interactions [93]. Solubilization of drug into the micelle can also alter the micelle hydrodynamic radius (Figure 6).

A popular variation of direct solubilization is the cold method, where poloxamer is dissolved in cold water (5–10 °C) along with the drug [104]. Poloxamers display a reverse solubility where they are more soluble at lower temperatures, as hydration of the block copolymer chains is strongest (Figure 2). As the temperature is increased, the solubility of the blocks decreases, especially the PPO block, resulting in microphase separated systems like micelles. The stronger state of hydration at low temperature provides faster dissolution times and should result in more homogenous mixtures, as unimers are prevalent. Changes in solubility of poloxamers with temperature variation have been exploited extensively in the formation of in situ hydrogel systems. Recent reports have explored poloxamer-based gelling systems using the cold method [105,106,107,108].

### 3.2. Thin Film Hydration

Another methodology for incorporating drugs into micelles is thin film hydration. In contrast to aqueous direct solubilization, organic solvents are typically used to create a solution of amphiphile and drug. Following a mixing step, the solvent is removed, typically with heat and vacuum, leaving behind a thin film. High encapsulation efficiencies are often observed in the thin film hydration method, since both the block copolymer and drug are initially dissolved in a common solvent. The resulting solid film of drug and amphiphile can later be rehydrated, again under heat and stirring, to form a micellar solution when desired. This is advantageous from a shelf-life perspective. Aqueous micellar systems can eventually deteriorate, meaning that long-term storage in such a format is not always desirable. A consequence of the evaporation step at elevated temperature is a dynamic change in the system composition. This can lead to structures intermediate between the micellar solution and the thin film states, such as hydrogels [109]. A variation on this methodology adds a lyophilization (freeze-drying) step, to remove lingering water or solvent without risking damage (i.e., from heating) to the structure of the block copolymer-drug matrix.

An early attempt of the thin film hydration approach was the solubilization of taxol by a diblock copolymer of poly(DL-lactide-co-methoxy polyethylene glycol) (PDDLA-MePEG) [110]. A number of solvents, including acetonitrile, chloroform, ethyl acetate and methanol, were evaluated, but only acetonitrile resulted in a clear, single-phase solution upon rehydration of the thin film. This is likely due to differences in solubility of the copolymer blocks and drug in various solvents, leading to the formation of different phases throughout the processing steps. This highlights the importance of solvent selection in the formulation steps. The solubility of taxol in this increased sharply with the diblock copolymer concentration and was higher for those with longer PDLLA blocks.

**Figure 7:**
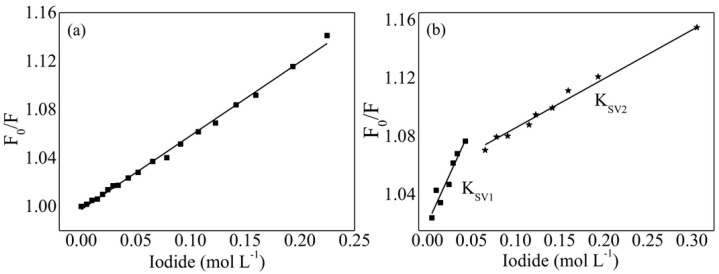
Stern–Volmer plots for (**a**) ERY-F127, where ERY is erythrosine, and (**b**) ERYBUT-F127, where ERYBUT is a butylated erythrosine ester derivative. Fo and F represent the fluorescence intensity of the photosensitizer in the absence and presence, respectively, of iodide, a water-soluble quencher. The samples were prepared by the ethanol thin film hydration method; micelles were re-dispersed by stirring with water at 60 °C for 8 h. In (**b**), two populations are observed. “Κ_SV1_” and “Κ_SV2_” correspond to ERYBUT located near the PEO (corona) and PPO (core) regions, respectively. Reprinted from [112]. Copyright 2016 Wiley.

A number of poloxamer-based drug formulations using thin film formation have since been reported. Redaporfin, a photosensitizer used in photodynamic therapy for cancer, was well-solubilized in thin film matrices of either Pluronic^®^ P123 or F127 [111]. The block copolymer and redaporfin were first dissolved together in ethanol and later hydrated in phosphate-buffered saline (PBS) to a concentration of 10% *w*/*v* block copolymer. The encapsulation efficiencies were 84% and 57% for P123 and F127 systems, respectively. The higher encapsulation efficiency in the case of P123 was attributable to stronger hydrophobic interactions between P123 and the drug, since P123 has a higher PPO fraction. Xanthene dyes of varying hydrophobicity have also been successfully incorporated into formulations containing 1% *w*/*v* P123 or F127 and 5 × 10^−6^ M dye via the thin film method [112]. A comparison of the direct solubilization and thin film hydration methods showed similar (and high) encapsulation efficiencies. However, the direct solubilization-prepared micelles began to release the xanthene dyes within days of preparation. On the other hand, rehydrated thin films displayed little-to-no measureable release over the duration of the study (30 days) [112]. This may be attributed to the difficulty experienced by the xanthene dyes to organize within the pre-formed micellar structure (in the case of direct solubilization, the xanthene was added to a micellar solution). During rehydration of the thin films, micellization and solubilization are expected to occur simultaneously. Additionally, the high concentration of drug in the film may lead to larger “micelles” upon rehydration, further entrapping the xanthene dyes. Interestingly, fluorescence quenching experiments meant to identify the location of drug within the hydrated structures showed bimodal distributions across the radius for some thin film method-prepared samples (Figure 7). The presence of two drug populations within the structure correlates with the hydrophobicity of the dye. Those dyes that are more hydrophilic are less likely to migrate completely to the PPO core [112].

Several recent publications that feature the thin film hydration method involve mixtures of different poloxamers [109,113,114,115]. A mixed poloxamer approach combines useful, typically opposing, properties. An example is the synergistic effect found in the solubilization of benzoporphyrin derivatives (BPD), a class of photosensitizers, into mixtures of Pluronics P123 and F127 [113]. At concentrations of 1% *w*/*v* and 5 × 10^−5^ M for block copolymers and BPD, respectively, encapsulation efficiencies between 89% and 95% were calculated for various BPDs and block copolymer ratios. Micelles composed of P123/F127 retained more photosensitizer in their cores upon 10- and 50-fold dilution with PBS than micelles of F127 or P123 alone. The longer, more hydrophilic F127 block copolymer molecules likely prevented stacking of cylindrical P123 aggregates [81], a drawback of the P123 block copolymer, which can offset the benefit of its strong solubilization. In general, mixed-block copolymer formulations with a mass ratio higher than 1:1 of hydrophobic to hydrophilic block copolymers achieved better encapsulation efficiencies [109,114]. The micelle shape, however, is impacted by the ratio of block copolymers [115], and especially by Pluronic^®^ P123, which tends to form cylindrical micelles.

A distinction should be made between equilibrium micelles produced via direct solubilization and the structures discussed in this section. The thin film hydration technique tends to produce micelle-like structures with drug loading, which may exceed that obtained under equilibrium conditions. Dynamic light scattering (DLS) measurements, which provide an intensity-weighted value of the size of dispersed structures, have provided evidence of bimodal distributions with indications of ill-defined aggregates. Redaporfin-Pluronic^®^ F127 mixtures resulted in structures around 25 and 110 nm, much bigger than equilibrium micelles, possibly indicating loose micelle aggregates, which could lead to ineffective administration of drug [111].

### 3.3. Temperature-Induced Emulsification

Drugs that are sparingly soluble in water can typically be dissolved in a suitable organic solvent to aid in the formulation. However, many of the added solvents are rather toxic and must be removed from the final product via an additional processing step. In order to address this need, moderate-to-high temperature emulsification techniques have been employed. One among these is hot melt extrusion [116,117], a technique that involves conversion of the drug from its crystalline to amorphous state in order to improve solubility during formulation. This typically involves raising the temperature well above the drug’s melting point, such that it is also above the glass transition temperature (T_g_) to ease the extrusion process. However, degradation of the drug at such high temperatures may occur.

The temperature-induced emulsification methodology utilizing poloxamers allows for lower processing temperatures. In this procedure, a given drug is mixed with a biocompatible solubilizing agent and then heated under mixing (or other method of homogenization) along with the poloxamer until the entire mixture is a homogeneous melt. The drug and solubilizing agent form an “oil” phase, which is encapsulated by the poloxamer. The melt is then rapidly cooled to about 0 °C to kinetically “trap” the emulsion. Upon rehydration, dispersed structures can be formed, the properties of which vary depending on which additives are used. Paclitaxel dissolved in low molecular weight poly(ethylene glycol) (PEG) was successfully encapsulated in Pluronic^®^ F68 by melting the ingredients together up to 120 °C and then rapidly cooling the homogenized mixture [118]. The F68/PEG ratio was 80%/20% *w*/*w*, so chosen based on the stability of Paclitaxel, which precipitated at >0.3 PEG/F68. The maximum stable drug load (ratio of the weight of drug solubilized in delivery vehicles to the total weight of the delivery vehicles) was 10% *w*/*w* Paclitaxel, with an encapsulation efficiency of 98%. Spherical particles of average diameter 101 nm were achieved at 7.5 *w*/*w* % Paclitaxel, but the polydispersity was high [118].

Multi-layer particles can be easily manufactured with this method, as well. By first creating a mixture of drug with Tween 80 (poly(ethylene oxide) sorbitan monooleate) and soybean oil, larger, lipid-like aggregates can be formed and then these encapsulated by poloxamer [119,120]. Larger spherical particles are obtained in these cases, with diameters in excess of 200 nm. Though the poloxamers make up the outer shell, the particle size is more strongly dictated by the drug-surfactant-oil core phase composition. DLS measurements of Pluronic^®^ F68-encapsulated Orlistat-Tween 80-soybean oil emulsions (prepared at 75 °C and then cooled) revealed a sharp decrease of the average particle size from about 370–240 nm as the Tween 80/soybean oil ratio changed from 0.1–1.0, with about ~230 nm being the minimum size observed across all formulations [119]. At the same, changing the Pluronic^®^ content from 50–80% resulted in slight and apparently random changes (~20 nm) in particle size, regardless of drug type, for a fixed composition of Tween 80/soybean oil. Other multifunctional components can be utilized, as well. Lupiodol^®^ was mixed with Tween 80 and 5 wt % Paclitaxel to form a core phase for encapsulation by Pluronic^®^ F68 for anticancer treatment [121]. The Lupiodol^®^ served as both an emulsifier for Paclitaxel and a tomography imaging agent.

The structures resulting from heat-induced emulsification are distinguished from thermodynamically-stable (equilibrium) micelles formed in the direct solubilization methodology. They are typically many times larger than poloxamer micelles, which are typically less than 40 nm. It is more accurate to consider these delivery vehicles as nanoparticles rather than micellar systems.

### 3.4. Solvent Displacement

Various solvent displacement techniques exist that rely on the mixture of fully-, partially- or non-miscible phases in order to spur the formation of polymeric drug vehicles. These include dialysis, direct solvent evaporation, emulsification-solvent evaporation and emulsification-solvent diffusion [122,123]. These methods are typically coupled with a lyophilization step in order to produce powders and, subsequently, pellets. Solvent evaporation followed by lyophilization has been successfully used to encapsulate Nonsteroidal anti-inflammatory drugs (NSAID) [124] and anticancer drugs [125,126] into poloxamer-based nanoparticles, which are powdered and pelletized. Simple dialysis has been used to separate drug-dissolving common solvents such as dimethyl formamide [127,128] and tetrahydrofuran [129] from conjugated poloxamer systems, leaving behind ready-to-use nanoparticle formulations.

Simple evaporation under mixing, heating, vacuum or combinations thereof has been prevalent in recent reports of poloxamer-based formulations [130,131,132,133]. Modulation of the particle size and drug encapsulation efficiency can be achieved by adding other polymers and surfactants to the solution in different proportions, creating aggregates or mixed micelles [134]. Aggregates of Pluronic^®^ F127 and the nonionic surfactant Solutol^®^ HS15 (a mixture of amphiphilic esters of 12-hydroxystearic acid) with the anticancer drug Icariside II were formulated with different HS15:F127 ratios. Increasing the ratio from 20:20–80:20 decreased the average aggregate size from 16.7–12.9 nm and increased the encapsulation efficiency from 88.4–94.8%. More significantly, the solubility of Icariside II was increased from 1.3–11.8 mg/mL as the relative amount of HS15 was maximized [135]. Oridonin-loaded aggregates with Soluplus^®^ (a polyvinyl caprolactam-polyvinyl acetate-polyethylene glycol graft copolymer excipient) and Pluronic^®^ P105 showed an increase in both diameter and encapsulation efficiency when changing the Soluplus^®^:P105 ratio from 4:1–3.75:1.25 and achieved a maximum drug loading of 15.1% [136]. Interestingly, decreasing the proportion of excipient resulted in larger mixed aggregates.

The emulsification-solvent evaporation technique relies on the ability of poloxamers to reduce the interfacial tension between oil-like and aqueous phases in order to produce drug-laden stabilized droplets. The typical procedure is to dissolve the drug and poloxamer in an organic solvent (frequently dichloromethane or ethyl acetate) and to mix this with an aqueous phase under high shear [122]. The organic solvent can subsequently be removed by evaporation, resulting in a solvent-free solution, which can optionally be lyophilized to produce powders [126,137,138,139,140]. The emulsion droplet size depends on surface tension and viscosity and so can be tuned by variation of the organic solvent type to influence the droplet surface area. The structure of micellar solutions of Pluronic^®^ P123 and Paclitaxel formed via the emulsification-solvent evaporation method using acetonitrile was studied by small-angle neutron scattering (SANS) at concentrations above the cmc [141]. The micelles formed were stable and well-described by a core-shell model, but the core radius (ranging from 34 to 37 Å) did not change significantly upon increasing the concentration of Paclitaxel despite the fact that the quantity of the solubilized drug increased [141]. The finding was attributed to Paclitaxel crystal growth as the acetonitrile was evaporated from the system (the solutions were centrifuged to remove large crystals). TEM imaging showed that P123 adsorbed along large Paclitaxel crystals (when they were not removed via centrifugation) [141].

### 3.5. Kinetically Frozen Micelles

Large excipient-to-drug ratios are typically required to achieve stability in the previously described methods, resulting in larger volume dosages, which can be inconvenient. Recently, an approach for preparing stable Poloxamer 407-based nanoparticles that are highly concentrated with drug has been reported [142,143]. Generally speaking, nanoparticles form when the micelle-to-unimer equilibrium is interrupted by a large energetic barrier to exchange (e.g., by the presence of glassy blocks or a selective solvent) [144]. The term “kinetically frozen” implies that the structure is trapped in a non-equilibrium state for long timescales relevant to the application.

**Figure 8:**
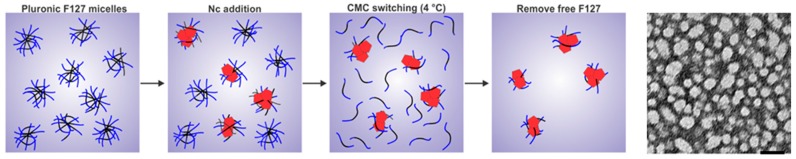
Preparation of kinetically-frozen surfactant-stripped drug-loaded nanoparticles. The process is initiated by dissolving the desired active ingredient in 10% *w*/*v* of Pluronic^®^ F127 (EO_100_PO_65_EO_100_) to produce drug-loaded micelles. As the temperature is decreased below the critical micellization temperature (to a temperature of 4 °C in this example), a majority of the self-assembled poloxamer chains dissociate away as unimers. The unimers are dialyzed out of the system, leaving behind poloxamer-stabilized nanoparticles [142].

The first step in the approach is co-micellization of the poloxamer and drug. After this, the temperature is reduced below the cmt, shifting the unimer-micelle equilibrium to free unimers from the drug-loaded micelles. The freed unimers are subsequently removed using dialysis, leaving behind nanoparticles with a high drug-to-polymer ratio. The cargo remains well dispersed below the cmt in the kinetically-frozen nanoparticles (Figure 8).

F127-naphthalocyanine frozen micelles of about 20 nm in size for intestinal imaging were generated using this “cold washing” technique [143]. Compared with a liposome-based formulation with the same initial amount of dye, a 50-times higher maximum absorbance in the near-infrared window was observed in the frozen micelles, termed nanonaps. Following the same methodology, vitamin K1 was encapsulated in poloxamer-stripped frozen micelles resulting in drug-to-poloxamer ratios of 50:1 and 25:1 in Pluronic^®^ F127 and F68-based formulations [142]. Several other amphiphiles were evaluated for potential use in this technique, but only Pluronics^®^ were capable of successful cmc-switching and subsequent formation of frozen nanoparticles. The addition of NaCl to Pluronic^®^-drug mixtures after surfactant stripping significantly improves the drug-loaded frozen micelle yield by making the solvent more polar and further promoting hydrophobic interactions between the Pluronic^®^ and drug [142]. Ten other biologically-relevant compounds were successfully loaded into kinetically-frozen micelles with drug:F127 molar ratios close to 50 (Table 3). The simplicity, diminished presence of potentially harmful additives and high drug-to-excipient molar ratios make this technique easily tailorable to many types of hydrophobic compounds. They may also allow higher dosing, thanks to the reduction in excipient content.

## 4. Conclusions and Outlook

Poloxamers are poly(ethylene oxide)-poly(propylene oxide) (PEO-PPO) block copolymers, which, due to their amphiphilic nature and unique self-assembly properties in aqueous solution, find use in a broad spectrum of fields. Their phase behavior depends strongly on the block composition and molecular weight, as well as the solvent conditions (temperature, presence of additives). The differing affinities of the constituent blocks allow for adsorption on solid and liquid surfaces. In the context of drug delivery, among the most useful features of poloxamers is their ability to solubilize hydrophobic drugs, thereby increasing bioavailability, and their thermoreversible gelation.

Drug delivery systems using poloxamers are amenable to multiple kinds of simple processing routes. These include: Direct solubilization, where simple aqueous or aqueous-organic solvent mixtures including drug and poloxamer result in encapsulation of the drug in poloxamer micelles; thin film methods, where drug-poloxamer mixtures are stabilized in a dried film structure, which can be rehydrated at the time of administration; and temperature-induced emulsification, where emulsions are formed at elevated temperatures and then rapidly cooled. The unimer-to-micelle transition (cmc switching) of poloxamers is also useful in the preparation of kinetically-frozen drug-loaded nanoparticles with high drug loading. The nanoparticles are stabilized by the presence of an energy barrier to the association or dissociation of unimers. The frozen state can be induced by the modulation of temperature or cosolvent concentration [145]. Many possibilities exist for the formulation of kinetically-frozen block copolymer micelles by judicious selection of the constituent blocks and processing solvent. In contrast, thermodynamically-stable (equilibrium) micelles are spontaneously formed via the hydrophobic effect. In the case of poloxamers, thermodynamically-stable micelles are less than 50 nm in diameter. These micelles are different from several other types of reported nanoparticles, aggregates, vesicles, etc., which can be hundreds of nanometers in size and which may not be thermodynamically stable. Structures that are thermodynamically stable such as micelles and hydrogels can be reversed by modulating the temperature (below the cmt or critical gelation temperature, respectively). Multi-component formulations that use poloxamers may be kinetically stabilized and typically undergo similar reversal of structure. In all of these cases, the amount of poloxamer in the final formulation is relatively low (typically around 20% *w*/*w* or less).

Targeted drug delivery is a major current thrust in the development of nanomedicines [146,147,148]. Improved efficacy and reduction in systemic side effects (e.g., from cytotoxic therapeutics) can be achieved by designing formulations to be more selective, stimuli-responsive and long-circulating. Poloxamers are promising for the formulation of nanomedicines due to their self-assembly and encapsulation properties [12,34,149]. Though not discussed in the present review, poloxamer-based formulations can take on several different physical forms within a relatively narrow window of concentrations and temperatures. The processing technique also controls the structure. Drug-loaded micelles of one or more type of poloxamer have shown promising results in the delivery of anticancer and benzoporphyrin derivatives for photodynamic therapy [113,150,151]. Mixed micelles and nanoparticles (the latter sometimes with structures of a diameter >50 nm) typically combine poloxamers of opposing hydrophilic-lipophilic balance (HLB) numbers in order to achieve increased solubilization (in the PPO core) and steric stabilization (PEO corona). At concentrations around 20% *w*/*w*, certain poloxamers will undergo a thermo-reversible phase transition around body temperature from micellar solution to a stiff gel (closely-packed cubic micellar form). Such systems are termed hydrogels and have been shown to be effective for the delivery of hydrophobic drugs [105,152]. The thermo-reversible nature of hydrogels allows the drug to be administered subcutaneously as a flowing micellar solution, which solidifies in situ to form a stable, long-lasting drug depot. Poloxamer-stabilized lipids have been used to produce lyotropic liquid crystals of bicontinuous cubic structure, which can be dispersed in water. The structures, termed cubosomes, have a high amount of hydrophobic and hydrophilic surface area, which can play host to drugs of varying HLB [153,154]. Multilayer “core-shell” structures have also been evaluated, including poloxamer-coated vesicles with encapsulated drug [155].

As alluded to earlier, poloxamer-based drug formulations are currently being investigated for the treatment of many diseases through various administration routes. Transdermal delivery of drug is convenient for patients, but penetrating the stratum corneum skin barrier is difficult. Poloxamers are well suited for this challenge due to their hydrophobic central block, which allows for interaction with lipids [156,157]. Their ability to adsorb on and penetrate oil films also makes them useful in ophthalmic delivery, where multiple obstacles lay. The hydrogel-forming characteristic of poloxamers is useful to counter constant tear generation of the eye, which can rapidly wash away the drug [158]. Oral administration of drugs can result in severe degradation because of bio-adhesion and passage through the digestive tract. Kinetically-frozen nanoparticles have been used to successfully image the intestine, giving high intensity images and no ill effect in a mouse model [143]. Hydrogel formation also allows for targeted subcutaneous drug delivery [159,160]. The influence of the structure of poloxamer-based vehicles on drug delivery in various administration routes draws connections between self-assembly, vehicle structure and resulting delivery mechanisms. Significantly, the insights gained from such research cut across many different drug and disease types.

## Figures and Tables

**Table 1:** Physicochemical properties of Pluronic^®^ PEO-PPO-PEO block copolymers often used in drug formulation. Data obtained from [40]. cmc, critical micellization concentration; L, liquid; P, paste; F, flake.

Pluronic^®^ Notation	MW	PO Units	EO Units	cmc at 25 °C (% *w*/*v*)	cmc at 30 °C (% *w*/*v*)	cmc at 35 °C (% *w*/*v*)
L64	2900	30	26	n/a	1.5	0.4
P65	3400	17	36	n/a	4	1
P84	4200	43	38	2.6	0.6	0.15
P85	4600	40	52	4	0.9	0.2
F88	11,400	39	206	n/a	n/a	1.7
P103	4950	60	34	0.07	0.01	0.002
P104	5900	61	54	0.3	0.04	0.008
P105	6500	56	74	0.3	0.025	0.005
F108	14,600	50	264	4.5	0.8	0.15
P123	5750	69	38	0.03	0.005	0.001
F127	12,600	65	200	0.7	0.1	0.025

**Table 2:** Physical properties [84] and structures [85] of compounds commonly studied for targeted drug delivery against ailments including cancer (paclitaxel), high cholesterol (fenofibrate), arthritic pain (ibuprofen and flurbiprofen), seizures (oxcarbazepine) and vitamin deficiencies (vitamin Κ1). The relative polarity of a drug molecule can be represented by its octanol-water partition coefficient (P), which is typically expressed as the logarithm of P, or LogP. Here, octanol exemplifies a hydrophobic environment.

Compound	Molecular Weight (g/mol)	LogP	Structure
Oxcarbazepine	252.3	1.5	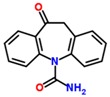
Paclitaxel	853.9	3.0	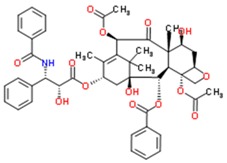
Ibuprofen	203.3	4.0	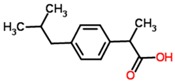
Flurbiprofen	244.3	4.2	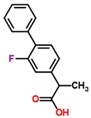
Fenofibrate	360.8	5.3	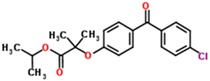
Vitamin K1	450.7	9.3	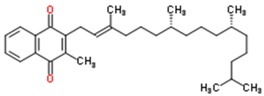

**Table 3:** Properties of kinetically-frozen surfactant-stripped drug-loaded nanoparticles of Pluronic^®^ F127. “Drug:F127 Molar Ratio” and “Drug Concentration” refer to the amount of drug solubilized into the nanoparticles, and the size refers to the nanoparticle dimensions. Data obtained from [142].

Compound	LogP	Drug:F127 Molar Ratio	Drug Concentration (mg/mL)	Size (nm)
α-Tocopherol	8.8	17	39	86
Cabazitaxel	3.7	8	41	62
Coenzyme Q10	9.9	30	43	82
Cholecalciferol	8.0	29	62	45
Cyclosporine A	4.1	15	7	165
Ergocalciferol	7.8	12	25	112
Ivermectin	4.4	45	80	39
Retinal palmitate	10.1	14	33	114
Squalene	8.6	44	80	81
Testosterone undecanoate	6.7	10	60	112
Vitamin Κ1	8.5	51	150	74
